# Phylogenomics of Unusual Histone H2A Variants in Bdelloid Rotifers

**DOI:** 10.1371/journal.pgen.1000401

**Published:** 2009-03-06

**Authors:** Karine Van Doninck, Morgan L. Mandigo, Jae H. Hur, Peter Wang, Julien Guglielmini, Michel C. Milinkovitch, William S. Lane, Matthew Meselson

**Affiliations:** 1 Department of Biology, University of Namur, Namur, Belgium; 2 Department of Molecular and Cellular Biology, Harvard University, Cambridge, Massachusetts, United States of America; 3 Laboratory of Immunogenetics, National Institute of Allergy and Infectious Diseases, National Institutes of Health, Rockville, Maryland, United States of America; 4 Laboratory of Bacterial Physiology and Genetics, Institute for Molecular Biology and Medicine, Université Libre de Bruxelles, Gosselies, Belgium; 5 Laboratory of Artificial and Natural Evolution, Department of Zoology and Animal Biology, University of Geneva, Sciences III, Geneva, Switzerland; 6 Harvard Mass Spectrometry and Proteomics Resource Laboratory, FAS Center for Systems Biology, Harvard University, Cambridge, Massachusetts, United States of America; 7 Josephine Bay Paul Center for Comparative Molecular Biology and Evolution, Marine Biological Laboratory, Woods Hole, Massachusetts, United States of America; Fred Hutchinson Cancer Research Center, United States of America

## Abstract

Rotifers of Class Bdelloidea are remarkable in having evolved for millions of years, apparently without males and meiosis. In addition, they are unusually resistant to desiccation and ionizing radiation and are able to repair hundreds of radiation-induced DNA double-strand breaks per genome with little effect on viability or reproduction. Because specific histone H2A variants are involved in DSB repair and certain meiotic processes in other eukaryotes, we investigated the histone H2A genes and proteins of two bdelloid species. Genomic libraries were built and probed to identify histone H2A genes in *Adineta vaga* and *Philodina roseola*, species representing two different bdelloid families. The expressed H2A proteins were visualized on SDS-PAGE gels and identified by tandem mass spectrometry. We find that neither the core histone H2A, present in nearly all other eukaryotes, nor the H2AX variant, a ubiquitous component of the eukaryotic DSB repair machinery, are present in bdelloid rotifers. Instead, they are replaced by unusual histone H2A variants of higher mass. In contrast, a species of rotifer belonging to the facultatively sexual, desiccation- and radiation-intolerant sister class of bdelloid rotifers, the monogononts, contains a canonical core histone H2A and appears to lack the bdelloid H2A variant genes. Applying phylogenetic tools, we demonstrate that the bdelloid-specific H2A variants arose as distinct lineages from canonical H2A separate from those leading to the H2AX and H2AZ variants. The replacement of core H2A and H2AX in bdelloid rotifers by previously uncharacterized H2A variants with extended carboxy-terminal tails is further evidence for evolutionary diversity within this class of histone H2A genes and may represent adaptation to unusual features specific to bdelloid rotifers.

## Author Summary

Bdelloid rotifers are microscopic animals common in ephemeral freshwater environments throughout the world. They are unusual not only because they have been reproducing without males for millions of years, but also because they can survive long periods of complete desiccation at any life stage and exposure to levels of ionizing radiation that cause hundreds of DNA double strand breaks per genome. Canonical histones (H2A, H2B, H3, and H4) are highly conserved proteins that package DNA in the nucleus and are involved in the regulation of chromatin metabolism. Because the conserved histone variant of canonical H2A, H2AX, is involved in the repair of DNA double strand breaks, we tested the possibility that bdelloid H2A histones are unusual. Strikingly, we find that bdelloids lack both H2A and H2AX, the absence of which is in contrast to their ubiquitous presence in other eukaryotes. Instead, we find that bdelloid rotifers replaced their canonical H2A protein by H2A variants not found in any other eukaryote. These results gain particular interest in view of the extreme resistance of bdelloid rotifers to desiccation and ionizing radiation and their attendant ability, possibly unique among metazoans, to repair massive levels of DNA breakage.

## Introduction

Rotifers of Class Bdelloidea are freshwater invertebrates of widespread occurrence that have attracted particular interest because their apparent lack of both males and meiosis suggests they are ancient asexuals [1]. Bdelloids are also of interest because of their extraordinary ability to survive and continue reproduction after desiccation at any life stage [2],[3] and doses of ionizing radiation that cause hundreds of DNA double-strand breaks (DSBs) per genome [4]. In contrast, rotifers of their sister class, the facultatively sexual monogonont rotifers, can survive desiccation only at a specific stage in their life cycle, as resting eggs [5],[6], and are not unusually resistant to ionizing radiation [4]. Since the H2A histones and some of their variants are well conserved in other eukaryotes and are involved in DSB repair and in certain meiotic processes [7], we speculated that bdelloid H2A histones may be unusual. In order to test this expectation, we investigated histone H2A genes and proteins of two species of bdelloid rotifers, *Adineta vaga* (*A. vaga*) and *Philodina roseola* (*P. roseola*), which represent two distantly related families within the Class.

Histones are architectural proteins that package eukaryotic DNA into nucleosomes, being essential in the maintenance, expression, and replication of the genome. The genes coding for the four canonical histones, H4, H3, H2B and H2A, which make up the nucleosome, are expressed during the S-phase of the cell cycle when the nuclear DNA is synthesized and are clustered in most metazoan genomes. These replication-dependent histone genes typically do not contain introns in animals and their mRNAs represent the only known cellular mRNAs that are not polyadenylated, ending instead in a highly conserved stem loop. The processing step in replication-dependent histone mRNA biosynthesis is a 3′-end endonucleolytic cleavage between the stem-loop and a so-called histone downstream element (HDE) [8]–[11]. In contrast, replication-independent histone genes encode variants of the canonical histones that are found outside of histone gene clusters and are expressed throughout the cell cycle. These variant histone genes may contain introns and their transcripts are polyadenylated [12].

Of the histone proteins, the H2A family includes the largest number of described variants and displays the greatest degree of diversity in carboxy-terminal tail length and sequence [12],[13]. One of these variants, H2AX, of wide occurrence in eukaryotes, is characterized by a unique and invariant C-terminal SQ(E/D)Φ-(end) motif, where Φ indicates a hydrophobic residue. It has been demonstrated, primarily in yeast and mammals [14],[15], that this highly conserved motif of H2AX is a consensus sequence for serine phosphorylation by PI3 kinases and that the serine residue is always located four amino acids from the C-terminal residue. In response to DSBs, H2AX becomes phosphorylated at this serine over large regions (∼2 Mb) surrounding the sites of breakage. Although the mechanistic implications of phosphorylation of the SQ(E/D) motif in H2AX are not fully understood, it is apparently required for normal DSB repair throughout the eukaryotic kingdom, being involved in the retention and accumulation of repair and checkpoint proteins to DNA breaks [for review], [ see 7], [14]–[19]. Moreover, it has been demonstrated in several eukaryotes that phosphorylated H2AX also plays a role in meiotic processes, including repair of meiotic DNA breaks made by SPO11 [20]–[23], prophase meiotic sex chromosome inactivation [23]–[25] and telomere movement [26]. With the exception of the nematode *Caenorhabditis elegans*, which lacks H2AX, it is ubiquitous throughout eukaryotes while the fruit fly *Drosophila melanogaster* has a H2AZ/H2AX chimeric H2A named *H2AvD* [16].

Considering the extreme radiation resistance of bdelloid rotifers [4] and the likelihood that, as in *Deinococcus radiodurans*, such resistance is an adaptation to repair and survive damage associated with desiccation including extensive DNA breakage [27],[28], one might expect in these organisms a high percentage of nucleosomal core H2A to be replaced by H2AX. For example in *Saccharomyces cerevisiae*, canonical H2A is replaced by H2AX and high levels of homologous recombination (and thus double strand breaks) occur [29]. Instead, we found that none of the H2A genes in the two bdelloid species have the H2AX-defining SQ(E/D) motif two amino acids from the C-terminal end and none of the bdelloid H2A genes is similar to canonical H2A. The absence of H2A and H2AX in bdelloid rotifers contrasts with their ubiquitous presence in other eukaryotes and with the presence of canonical H2A in the monogonont rotifer *Brachionus plicatilis (B. plicatilis)*. The three different types of H2A genes we found in bdelloid rotifers are apparently unique to bdelloids and form distinct lineages that evolved from canonical H2As. We also found that the regions of the genome containing histone gene clusters are organized as two co-linear pairs, consistent with the degenerate tetraploidy of bdelloid rotifers [30],[31], with one pair lacking an H2A gene in the cluster while the other cluster contains an H2A gene, designated *H2Abd*, that has an unusual C-terminal tail. Although present in a cluster containing the canonical H4, H3 and H2B genes, it is not *H2Abd* that is highly expressed under normal conditions in the nucleosomes of both bdelloid species; instead, the principal H2A found in the nucleosomes is an unusual H2A variant coded by a gene designated *H2Abd1* that is not located in the histone gene cluster.

It seems reasonable to speculate that the various unusual features of bdelloid H2A histones are associated with the adaptation of bdelloids to survive desiccation and perhaps also with their lack of meiosis.

## Results

### Genomic Library Screens

**Figure 1:**
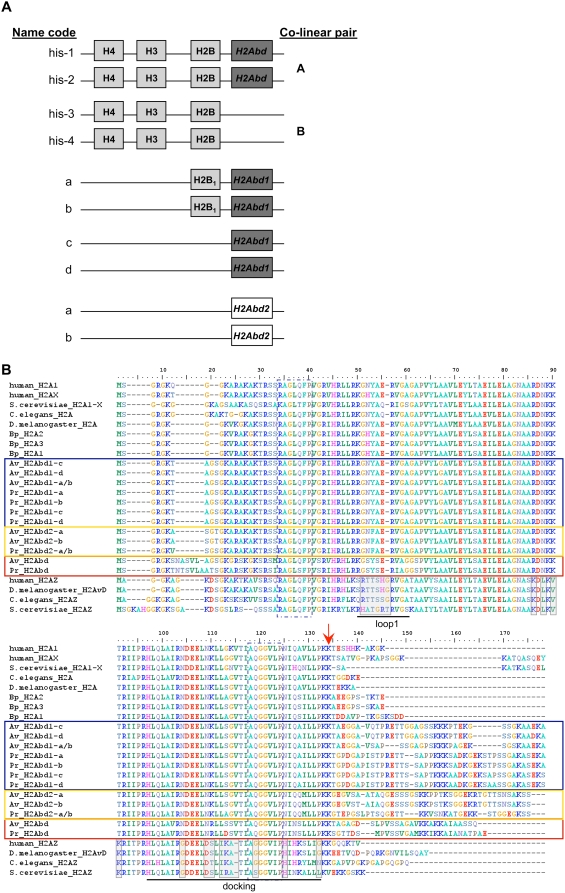
Unusual histone H2A variants of the bdelloid species *Adineta vaga* (Av) and *Philodina roseola* (Pr). (A) All three bdelloid H2A variants (*H2Abd*, *H2Abd1* and *H2Abd2*), with their respective name code for each copy, are given. Each copy of the histone gene cluster is also represented, being organized as co-linear pairs A and B. Presented in light grey boxes are genes likely present in the nucleosomes; dark grey boxes represent expressed variants found in the bdelloid proteome (see below); white boxes represent unknown expression status. (B) Multiple alignment using MAFFT v6 of the histone H2A genes of the bdelloid species Av and Pr and the monogonont rotifer *Brachionus plicatilis* (Bp) with canonical H2A, H2AX and H2AZ variants of few other model eukaryotes. Red, blue and yellow boxes indicate the bdelloid *H2Abd*, *H2Abd1* and *H2Abd2* genes, respectively. The red arrow indicates the start of the C-terminal tail beyond the conserved LLPKK motif. Protein interaction domains (“loop1” and “docking”) are indicated and taken from [37]. The regions of primer design are in a dashed box and the light grey boxes represent the regions where H2AZ differs substantially from canonical H2A.

Primary genomic fosmid libraries of *A. vaga* and *P. roseola* were separately probed with PCR amplification products obtained by using primers based on highly conserved regions of the canonical H3 and H2A histone genes (indicated in Figure 1B). Individual fosmids hybridizing to both probes should contain the clustered histone H3 and H2A genes while fosmids hybridizing only to the *H2A* probe would be expected to contain the *H2AX* variant or any other non-clustered H2A variant that may be present and which, like H2AX, has a primary sequence similar to that of canonical H2A. Fosmids that hybridized to either or both probes (∼120 in *P. roseola* and ∼225 in *A. vaga*) were tested by PCR and by direct sequencing, leading to the isolation of ∼80 fosmids from *P. roseola* and ∼180 fosmids from *A. vaga* containing histone H3 and H2A genes.

All fosmids from both bdelloid species containing canonical H3 fell into one of four categories, each coding for the same highly conserved H3 amino acid sequence (Figure S1), but clearly distinguishable at synonymous sites. One fosmid of each category from both *A. vaga* and *P. roseola* was fully sequenced (∼35 kb) and annotated giving contigs Avhis-1 (EU652315), Avhis-2 (EU850438), Avhis-3 (EU652316), Avhis-4 (EU850439) and Prhis-1 (EU850440), Prhis-2 (EU652317), Prhis-3 (EU652318) and Prhis-4 (EU850441) respectively (Figure 1A, Figure S1) [see also 30]. Two of these fosmids from each species, designated co-linear pair A, contain genes for the canonical histones H4, H3, H2B and a variant of H2A, and are highly similar. Fosmids of the other pair found in both species and designated co-linear pair B, are also highly similar to one another but lack the H2A gene (Figure 1A, Figure S1). Pair B is considerably diverged from pair A (Ks *ca* 45 percent) and some non-histone genes present in each pair are not present in the other. This pattern is consistent with the degenerate tetraploidy of bdelloid genomes [30],[31]. The H2A gene found in half of the histone clusters in both *A. vaga* and *P. roseola* and designated *H2Abd*, has a long and unique carboxy-terminal tail with a sequence unlike the C-terminal tail of any canonical or variant H2A known in other metazoans (Figure 1B, Genbank accession numbers for the two nucleotide copies in *A. vaga* EU853686, EU853685 and in *P. roseola* EU853693, EU853694).

Other fosmids in both bdelloid species were found to contain histone H2A genes not in clusters, but also with a uniquely long carboxy-terminal tail and are designated variants *H2Abd1* and *H2Abd2*. The H2A gene *H2Abd1* is present in four copies in both *A. vaga* and *P. roseola*, in two co-linear pairs of contigs, only one of which includes a gene for H2B (Figure 1A) and with about 50 percent synonymous divergence between gene copies in different pairs, again consistent with degenerate tetraploidy [30],[31] (Genbank accession numbers for the nucleotide copies a, b, c, d in *A. vaga* EU853687 to EU853690 and in *P. roseola* EU853695 to EU853698). *H2Abd2*, the third H2A variant, also found in both bdelloid species, is not clustered with any histone gene, and is present only as two closely similar copies within a co-linear pair of contigs (Figure 1A, Genbank accession numbers for the two nucleotide copies a and b in *A. vaga* EU853691, EU853692 and in *P. roseola* EU853699, EU853700).

The bdelloid histone H2A variants and the canonical H2A genes we found in the monogonont rotifer *B. plicatilis* are aligned along with the canonical H2A genes and H2A variants of other eukaryotes in Figure 1B. Only the variants H2AX and H2AZ, found in most eukaryotic lineages are represented in the alignment. The macroH2A and Barr-body deficient H2A (H2A Bbd) variants are not included because they are vertebrate-specific [13]. All three types of bdelloid H2A genes, *H2Abd*, *H2Abd1* and *H2Abd2*, code for C-terminal amino acid sequences extending 28–43 amino acid residues beyond the canonical LLPKK motif and are typically longer than those found in other metazoans, with the exception of human macroH2A (Figure 1B and Table S1). Interestingly, none of the bdelloid-specific H2A C-terminal tails resemble those of other canonical H2As represented in Genbank and all lack the SQ(E/D)Φ-(end) motif characteristic of H2AX, indicating that both of these highly conserved proteins are absent from bdelloid rotifers.

Since all H3 and H2A-containing fosmids were examined and the same three H2A variant genes, in the same organization, were found in both *A. vaga* and *P. roseola*, it is likely that we have identified all copies of the H2A genes containing a canonical H2A core. The canonical H2A genes we found in the monogonont rotifer *B. plicatilis* closely resemble the canonical H2A genes present in most eukaryotes and differ substantially from the bdelloid-specific H2A variants (Figure 1B). The presence of all three H2A variants in species representing two different bdelloid families [32] but not in the monogonont suggests that they are characteristic of the entire class Bdelloidea and have arisen after the separation of bdelloids and monogononts but before the bdelloid radiation.

### Histone Proteins

**Figure 2:**
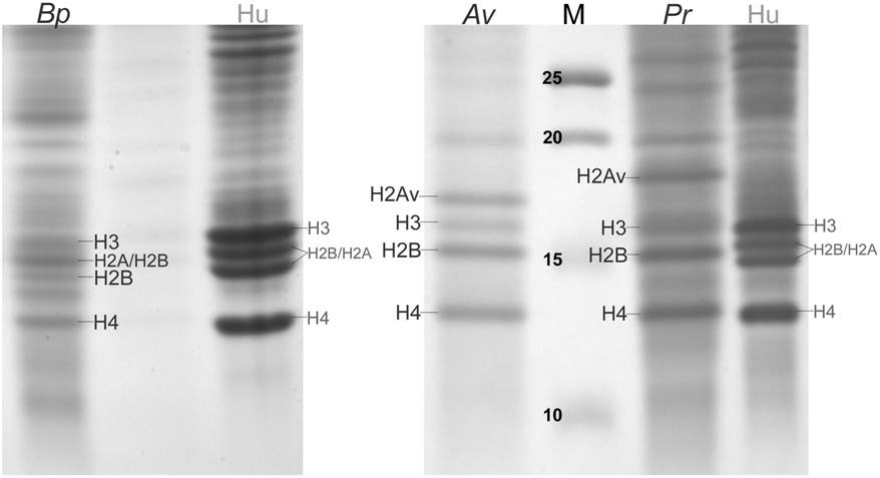
SDS-PAGE gels of bdelloid histone proteins. The histone proteins extracted from the nuclei of *Adineta vaga* (*Av*), *Philodina roseola* (*Pr*), *Brachionus plicatilis* (*Bp*) and human HeLa cells (Hu) are represented on SDS-PAGE gels. All the bands visible on the gel up to ∼25 kD for both bdelloid species and till ∼20 kD for *B. plicatilis* were identified by mass spectrometry: only histone bands are labelled here. The numbered bands in lane M (marker) represent the protein size ladder in kD.

In order to confirm the absence of canonical histone H2A, and to determine which histone H2A replaces it in the nucleosomes of *P. roseola* and *A. vaga*, we compared the histones in the nucleosomal fraction of both bdelloid species with those of the monogonont rotifer *B. plicatilis* and human HeLa cells by denaturing gel electrophoresis (SDS-PAGE - Figure 2) and liquid chromatography-tandem mass spectrometry (LC-MS/MS) of peptides following enzymatic digestion.

Except for the bdelloid H2A, all rotifer histones displayed an electrophoretic mobility indistinguishable from to that of their human homologs. The identity of rotifer histones was further verified by LC-MS/MS, confirming the presence of the canonical histone proteins H4, H2B and H3 in bdelloid nucleosomes and all four canonical histones in monogononts. The region of the gels between rotifer H4 and H2B and the three prominent bands of mass greater than that of rotifer H3 (Figure 2) were also examined by LC-MS/MS. Only the first prominent band of greater mass than H3 from each bdelloid proved to be a histone (band H2Av in Figure 2). This band was identified as *H2Abd1* by mass-spectrometric analysis of carboxyl terminal tails. Such detailed analysis also identified peptides coded by each of the four copies of the gene *H2Abd1* in both bdelloids as well as a minor quantity of peptides corresponding to *H2Abd* from *A. vaga* (an example for *H2Abd1* of *P. roseola* is given in Figure S2). No peptides from either bdelloid species coded by *H2Abd2* were detected by LC-MS/MS.

The substantial mass difference observed in Figure 2 between band H2Av of *A. vaga* and that of *P. roseola* is consistent with the difference in C-terminus tail length and the calculated mass for proteins coded by *H2Abd* and *H2Abd1* in *A. vaga* and *P. roseola* (Table S1).

The results of the SDS-PAGE/LC-MS/MS analysis corroborate the findings from the genomic sequence data: canonical H2A is absent from both bdelloid species and is replaced by the variant *H2Abd1* and, at a much lower level, by *H2Abd*.

### Histone Gene Characteristics

**Figure 3:**
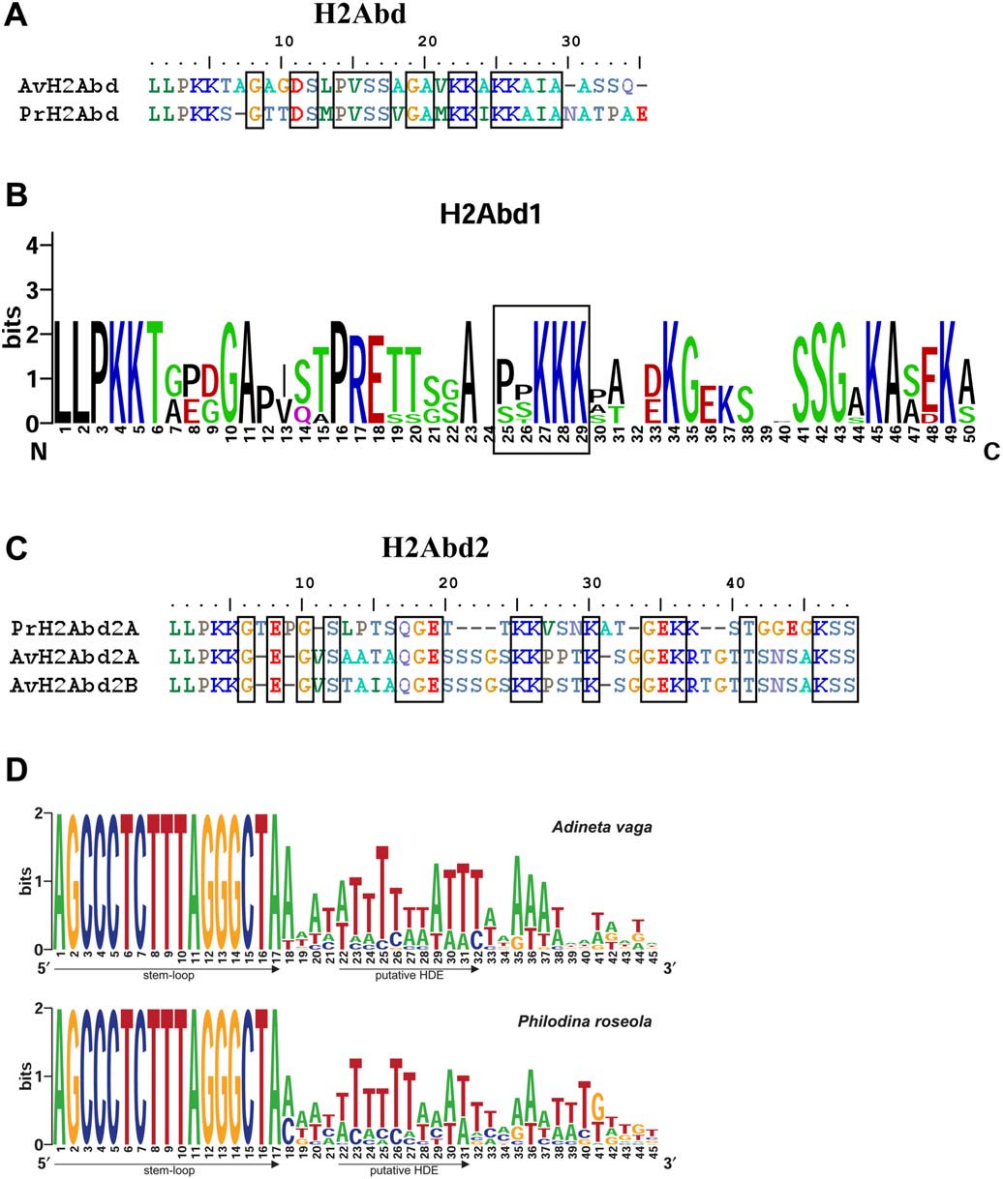
Alignments of the bdelloid-specific H2A carboxy-terminal tails and nucleotide stem-loop sequences. (A) Alignment of the *H2Abd* C-terminus tails of *Adineta vaga* (Av) and *Philodina roseola* (Pr); conserved amino acids are framed. (B) Alignment in Logos format of the *H2Abd1* C-terminal tails of Av and Pr; a putative SPKK class of motif is represented in a box. (C) Alignment of the *H2Abd2* C-terminus tails of Av and Pr; conserved amino acids are framed. (D) Sequence Logo representation of the conserved 3′ UTR stem-loop and downstream sequence (45 bp) of all histone genes in Av and Pr (except *H2Abd2* that does not exhibit a stem-loop). UTR, untranslated region.

There is little similarity in amino-acid sequence among the three different H2A variant C-terminal tails in a given bdelloid species (Figure 1B). There is also considerable interspecies amino-acid difference between C-terminal regions of the same variant although a few short motifs are conserved. The C-tails of the histones coded by the four copies of *H2Abd1* in both *A. vaga* and *P. roseola* were aligned and represented in Logos format with the ‘tallest’ residues representing the most conserved amino acids (Figure 3B). Within this C-terminal tail the framed block resembles a putative four-residue long S(T)PK(R)K(R) class of minor groove DNA binding motifs in which P has a strict position at i^+1^ [33]. A variety of these SPKK motifs have been found in termini of histone H1 and in the N-terminal tail of sea urchin histones H2B [34],[35] but also in the N-terminal tail of *Drosophila* centromeric H3 [33]. Until our study, its presence in the C-terminal of H2A was known only in plants [36]. In all these instances the SPKK motifs mediate histone interactions with linker DNA in the minor groove. The *P. roseola H2Abd1* C-terminal tails contain such a motif while in *A. vaga* a variation of it appears to be present (Figure 3B).

The amino acid alignment of the two copies of both *H2Abd* and *H2Abd2* in *A. vaga* and *P. roseola* is given instead of the Logos representation (Figure 3A, 3C). Although no similar SPKK class of DNA binding motifs was found in these variants, there are several conserved amino acids.

Excluding the distinctive C-terminal tails (starting at the arrow in Figure 1B), the bdelloid H2A genes all have an amino acid sequence closely similar to that of the canonical eukaryotic H2A. The H2A residues involved in histone-histone interactions, such as those of loop 1 that interacts with the other histone H2A and those of the docking domain that contacts the H3-H4 dimer, are well conserved between the three bdelloid H2A variants and canonical H2A (Figure 1B). Such conservation is also characteristic of H2AX, while all other H2A variants differ substantially from the canonical H2A [29],[37]. H2AZ diverges specifically in three different regions including the above histone-histone interaction zones (see Figure 1B). The alignment of Figure 1B suggests that the three unusual bdelloid H2A variants are closely related to canonical H2A over the entire histone fold domain (see also the phylogenetic analysis below).

We also examined the characteristics of the DNA sequence beyond the stop codon of the different bdelloid histone H2A variants and the canonical H3, H4 and H2B genes. Typically, only the replication-dependent histone genes expressed during DNA synthesis have a characteristic, unique 16-nucleotide stem-loop sequence in the 3′ untranslated region [8]–[11]. In contrast, all of the bdelloid histone genes depicted in Figure 1A except *H2Abd2* have the same 16-nucleotide stem-loop sequences 40–80 bp beyond the stop codon (Figure 3D and Table S1). The stem loop is similar to those in other metazoans, consisting of a four-nucleotide loop (CTTT) and a six base-pair stem (Figure 3D). In addition to the 16 nucleotides of the stem-loop structure, there is a high degree of conservation of the ten nucleotides before the stem (not shown). There is also a conserved AT-rich region at the position of a putative histone downstream element (HDE), 4–12 bp downstream of the stem-loop in all of the histone genes of *A. vaga* and *P. roseola* except *H2Abd2* (Figure 3D). A similar region is present in the replication-dependent histone genes of *C. elegans* and is believed to be involved in histone 3′ mRNA processing [11]. Both H2A variants *H2Abd* and *H2Abd1* in both bdelloid species contain the stem-loop motif beyond the stop codon, as well as a putative polyadenylation signal similar to that of H2AX. This latter variant, ubiquitous in other eukaryotes but absent in bdelloids, is packaged in nucleosomes during DNA-replication and is also deposited preferentially in response to DNA double strand breaks [38]. *H2Abd2* does not contain this stem-loop motif but has a putative polyadenylation signal.

Another unusual feature of bdelloid histones is the presence of introns in each of the various histone genes (canonical histones and variants) in one or both bdelloid species, except for the H2B gene adjacent to *H2Abd1* (Table S1). The length of the introns ranges from 52 to 76 bp, which is typical of bdelloid introns [39]. This contrasts with the absence of introns in the canonical replication-dependent histone genes in other animals, although they are present in the canonical histone genes of plants and fungi [40],[41].

### Adaptive Evolution

**Figure 4:**
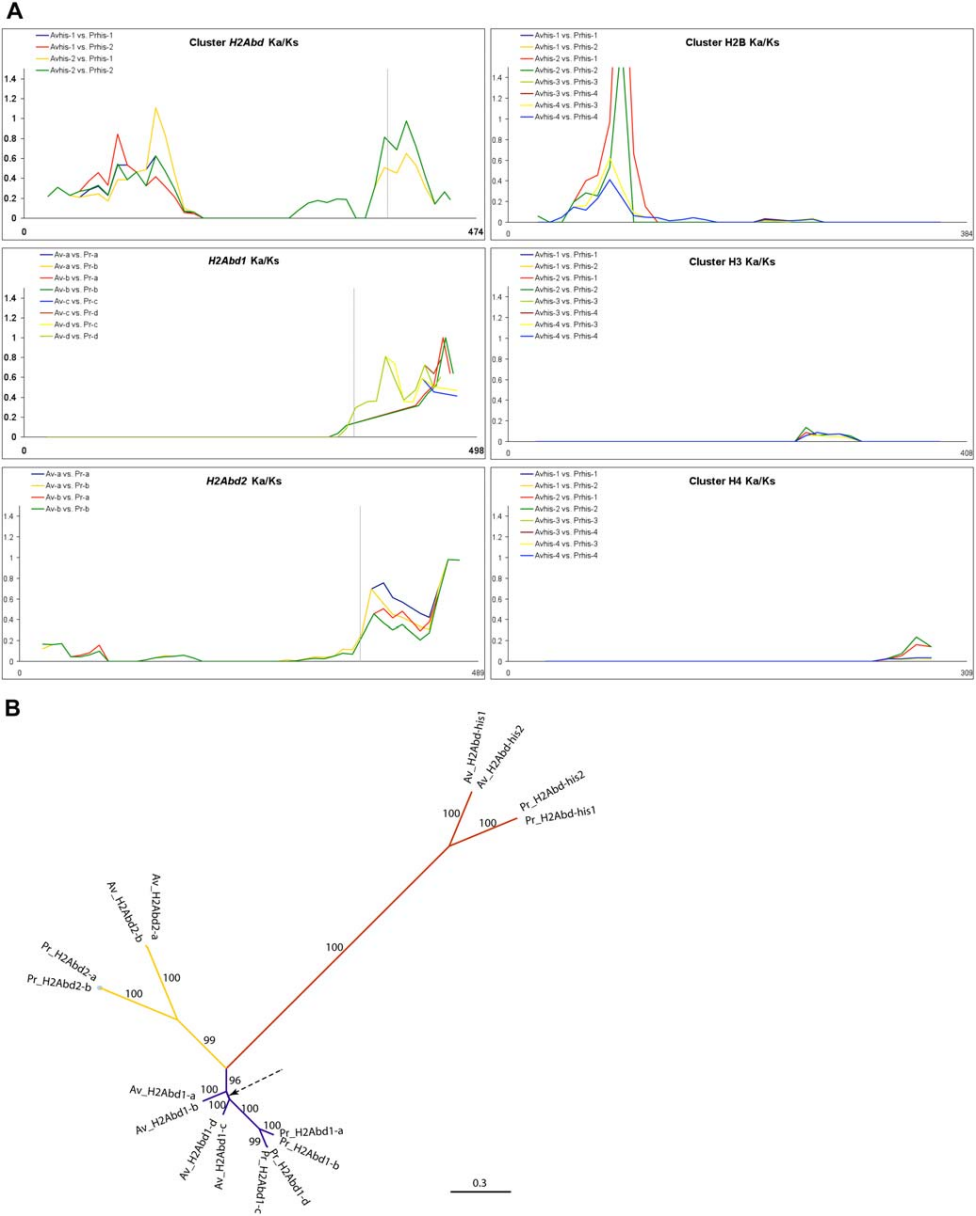
Molecular evolution of bdelloid-specific H2A variants. (A) Ka/Ks ratios of the bdelloid rotifer histone genes: inter-species, intra co-linear pair comparisons between *Adineta vaga* (Av) and *Philodina roseola* (Pr). The line present in the 3 graphs of the first column represents the start of the C-terminus tail beyond the LLPKK motif in H2A genes. (B) Maximum Likelihood phylogram of bdelloid H2A gene variants inferred with RaxML. Bootstrap supports >50% are shown for each node. The arrow indicates the branch with bootstrap <50%.

Inter-species comparisons of the ratio of amino acid changes to synonymous changes (Ka/Ks) in the bdelloid histone gene sequences indicate that the amino acid sequences of the clustered H2B, H3 and H4 genes (Figure 4A, right column) are highly conserved except for 2 amino acids in the N-terminal region of H2B. Such conservation is characteristic of eukaryotic canonical histones. The H2A variants *H2Abd*, *H2Abd1* and *H2Abd2* are also highly conserved, with the exception of their C-terminal tails and a short region in the N-terminal region of *H2Abd* (Figure 4A, left column).

In order to detect and measure selection on bdelloid rotifer H2A proteins we used the programs SELECTON [42],[43] and PAML [44],[45]. For both programs we used an amino acid based nucleotide alignment and corresponding phylogenetic tree (tree represented in Figure 4B). The H2A histone fold domains of the different bdelloid H2A variants, but not their C-terminal tails, are under strong purifying selection as detected by SELECTON (results not shown) and also seen in Figure 4A. The program PAML was used to search for signal of positive selection in the bdelloid H2A genes; it implements a likelihood ratio test for positive selection based on dN/dS rate ratios [46] on specific branches [47], on individual codon sites [48], or on both simultaneously (*i.e.*, a branch-site method for testing positive selection on individual codons along specific lineages) [49]. No significant branch specific selection was found. The PAML models used for detecting site-specific selection were M1a and M7 for neutral evolution and M2a, M8 for positive selection. All models were significantly better than model M0 indicating that the dN/dS ratio varies along the sequence but no significant positive selection was detected, as the comparisons M1a-M2a and M7-M8 were not significantly different.

The phylogenetic tree represented in figure 4B clusters the two bdelloid species for each specific H2A variant. It therefore appears that the different H2A variants arose before the separation of the two bdelloid families and have been diverging since mostly through neutral evolution (as detected by SELECTON, results not shown).

### Phylogenetic Analysis

**Figure 5:**
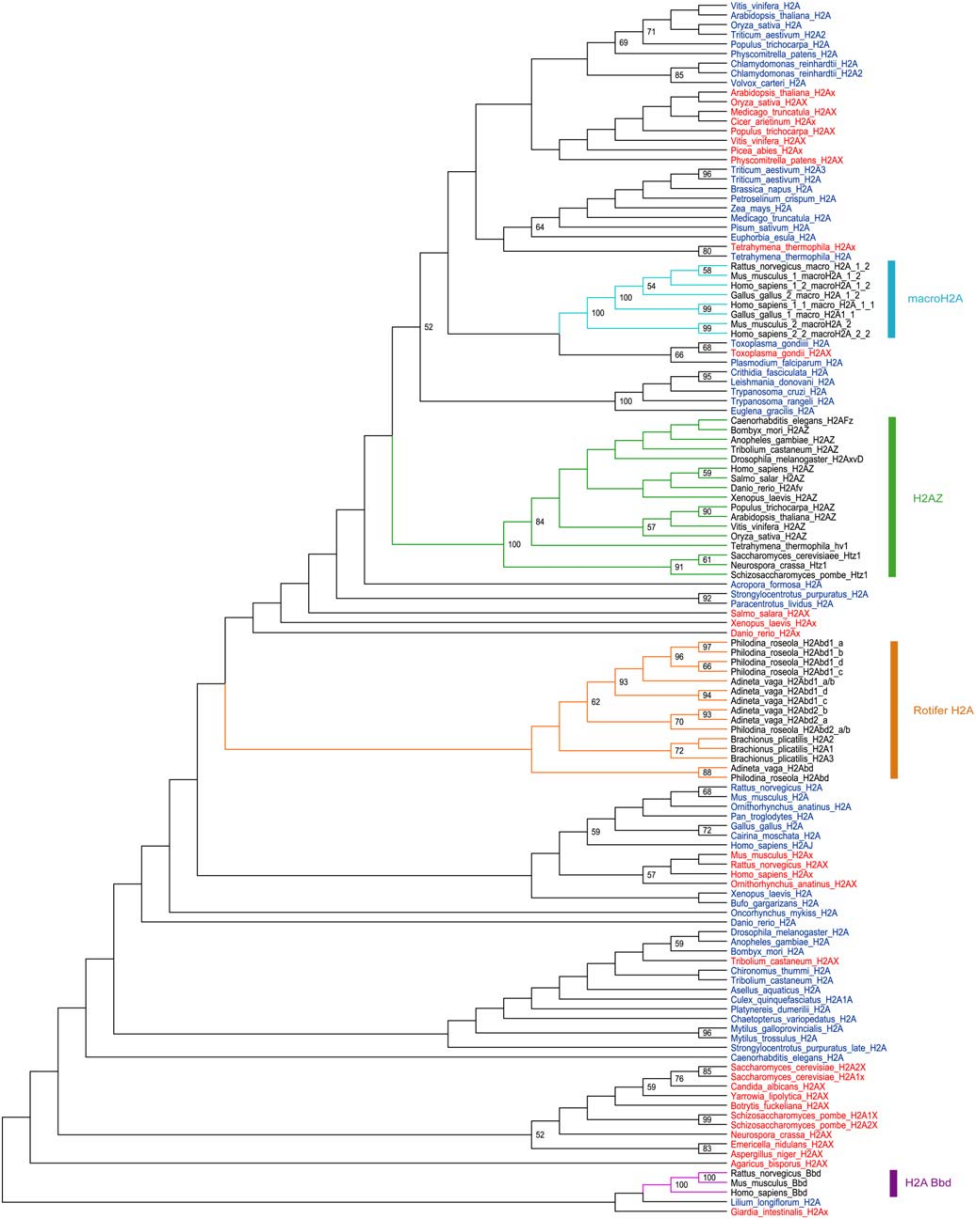
Phylogeny of H2A proteins. Maximum likelihood bootstrap consensus cladogram, inferred using the program Bootstrap RaxML, of canonical H2A and variants. Bootstrap values >50% are shown. Variants are colour-coded and all canonical H2As are in blue and H2AX in red. All H2A bdelloid rotifer genes form, with canonical H2As of *Brachionus plicatlis*, a distinct lineage (orange) nested within the eukaryotic canonical H2As.

A multiple amino acid alignment of canonical H2A genes and H2A variants was carried out in MAFFT [50],[51] (result not shown). The H2A variants included in this alignment are H2AX and H2AZ from distinct eukaryotic lineages, the vertebrate-specific H2A variants macroH2A and Barr-body deficient H2A (H2A Bbd), and the bdelloid H2A variants (*H2Abd*, *H2Abd1* and *H2Abd2*). Based on this alignment we inferred the phylogeny of the H2A proteins using a maximum likelihood approach as implemented in Bootstrap Raxml [52] (Figure 5). Due to the highly different types of H2A genes included (belonging to different eukaryotic lineages), several parts at the root of the tree are unresolved but some distinct groups are apparent. Congruent with previous phylogenies [16],[29] the vertebrate-specific variant H2A Bbd (purple) forms a distinct cluster outside the canonical H2A group while vertebrate MacroH2A (light blue) is a distinct lineage within canonical H2As. The H2AZ variants (green), having a role in transcription [13] and thought to be universally conserved, clearly form a monophyletic group distinct from canonical H2A. The evolution of the H2AX gene (red) is different from the other H2A variants because it had multiple evolutionary origins within the eukaryotic kingdom as concluded previously [16],[29] and entirely replaced canonical H2A in fungi and *Giardia* (Figure 5). The H2A variants of the bdelloid rotifers (orange) form a distinct lineage, with *H2Abd*, *H2Abd1* and *H2Abd2* clustering with the canonical H2A genes of the monogonont rotifer *B. plicatilis* (Figure 5). This analysis demonstrates that the bdelloid-specific H2A genes are not closely related to any of the other H2A variants but evolved from a canonical H2A of a common monogonont-bdelloid ancestor.

**Figure 6:**
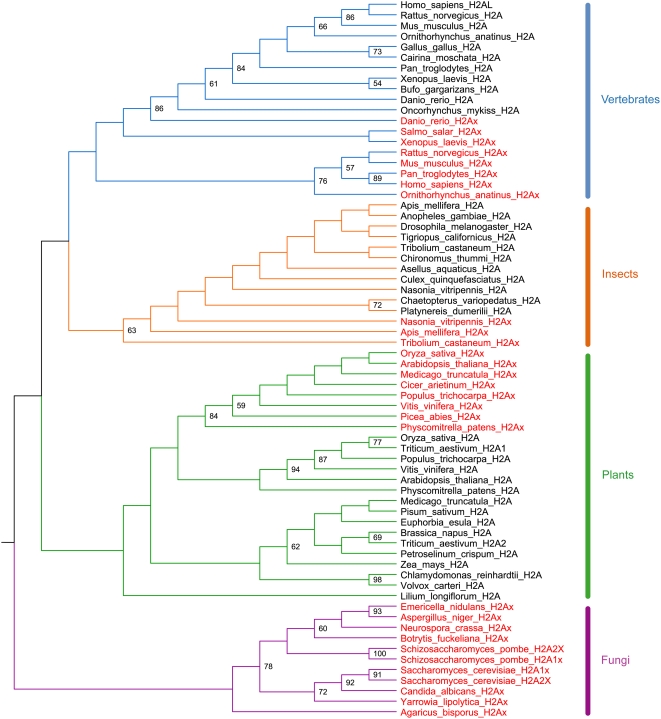
Phylogeny of canonical H2A and H2AX proteins. Maximum likelihood bootstrap consensus cladogram, inferred using the program Bootstrap RaxML, of canonical H2A and H2AX of fungi, insects, plants and vertebrates. Bootstrap values >50% are shown. Eukaryotic groups are colour-coded; H2AX is in red and canonical H2As in black.

We investigated in more detail the convergent evolution of H2AX found here and also by Li et al. [16] and Malik&Henikoff [29] by repeating the phylogenetic analysis in bootstrap Raxml using only H2A and H2AX sequences of specific plant, insect, vertebrate and fungi species in order to obtain a better alignment than in the previous analysis. It appears from this phylogeny (Figure 6) that the H2AX variants evolved multiple times but at a higher-order level than indicated in the previous phylogenies (Figure 6) [16],[29]. Indeed, the H2AX variants cluster together rather then with canonical H2As within the plants, vertebrates and insects and, hence, have a single origin within each of these groups.

## Discussion

While the bdelloid canonical histones H2B, H3 and H4 are highly similar to their counterparts in other eukaryotes, we find that the bdelloid complement of H2A histones is highly unusual, with carboxy-terminal tails that are much longer than those of canonical H2A and that are unlike any other eukaryotic H2A variants. The bdelloid H2A histones may be classified as heteromorphous variants because the extent of amino acid sequence change involves a large portion of the C-terminal tail and not merely a few changes as is typical of H2A isoforms [13]. Even the H2A gene *H2Abd* found in half of the histone clusters in both bdelloid species codes for an unusual C-terminal tail and it seems apparent that canonical H2A is absent from bdelloid rotifers. One of the bdelloid variants, the unclustered *H2Abd1* gene, is highly expressed in the embryos of both species during normal growth, while expression of the clustered *H2Abd* gene was detected at a substantially lower level and only in *A. vaga*. The *H2Abd* and *H2Abd1* sequences in both bdelloid species specify both the stem-loop motif characteristic of the replication-dependent histones in other eukaryotes and a putative polyadenylation signal in the 3′ UTR characteristic of replication-independent histone variants. Since both of these bdelloid-specific H2A variants H2Abd and H2Abd1 have a stem loop beyond the stop codon and were found in the nucleosomal fraction, they are probably the H2A proteins incorporated into nucleosomes during normal DNA replication in bdelloid embryos, the only life stage in which mitosis occurs in the somatic cells of these eutelic organisms. These bdelloid H2A variants may also be expressed at other times throughout the cell cycle, as observed for H2AX in other eukaryotes [38], while H2Abd2 variants in both species lack the stem-loop motif and the corresponding histones were not found in the protein analysis of nucleosomal fractions. Nevertheless, considering the strong purifying selection under which they have evolved, the histones coded by these variants are almost certainly expressed and incorporated into nucleosomes under some specific conditions. The monogonont rotifer *B. plicatilis*, belonging to the sister class of bdelloids, has canonical H2A and appears to lack variants similar to those present in bdelloids.

Since the three bdelloid H2A variants are found in each of the two studied species belonging to distantly related families, and as the bdelloid H2As group together with the canonical H2A of monogonont rotifers in the H2A phylogenetic tree (Figure 5), it seems likely that all these variants evolved from a canonical H2A ancestor. The evolutionary process that took place involved the alteration and extension of the H2A carboxy-tail by at least 25 amino acids and the appearance of conserved motifs. One of these motifs, found in the variant expressed in the nucleosomes (*H2Abd1*), seems related to the S(T)PK(R)K(R) class of DNA-binding motifs and may play a role in the interaction with linker DNA and the packaging of the nucleosome. Moreover, the protein extension of the H2A C-terminal tail is in a region of the nucleosome close to the binding site of histone H1 and hence may affect the structure or dynamics of the nucleosome [12],[16]. Indeed, H2A constitutes the largest heterogeneous family of histone variants that are active in distinct aspects of chromatin conformation and genomic function and the results presented here are consistent with the evolutionary diversity within the H2A family. The high degree of sequence conservation observed within the histone fold domains of the different bdelloid H2A variants is consistent with the general finding that H2A variability is largely confined to the carboxy-terminal domain, both in length and composition [12]. The inter-species variability found in the carboxy-tails of each bdelloid H2A variant (Figure 3A,B,C) could be the result of neutral evolution after the separation of the two families, as seen in the SELECTON analysis, and may suggest that the extension has a more significant role than its particular amino-acid sequence.

By analogy with the radiation- and desiccation-resistant bacterium *Deinococcus radiodurans*, in which prolonged desiccation causes extensive DNA breakage [27],[28], it is likely that bdelloid radiation resistance similarly reflects an adaptation to survive DNA breakage associated with the frequent desiccation events they experience in the ephemerally aquatic habitats they typically inhabit [4]. DNA breakage in other eukaryotes is accompanied by phosphorylation at the serine in the invariant S^[−4]^Q(E/D)(I/L/F/Y) motif found in H2AX of protists, fungi, plants and animals. In fungi and *Giardia*, the H2AX variant has completely replaced the canonical H2A. Considering the involvement of H2AX in the cellular response to DSBs and its ubiquitous occurrence in eukaryotes, we expected to find H2AX genes and a high percentage of H2AX proteins in bdelloid nucleosomes. Instead, none of the bdelloid H2A genes contain the S^[−4]^Q(E/D) motif characteristic of H2AX. Although SQ occurs at the final two residues of *A. vaga H2Abd* and 26 amino acids from the C-terminal end of *P. roseola H2Abd2*, it is notable that these SQ residues are present in a different H2A variant in each species. They are therefore not conserved across bdelloid families and may therefore not represent a functional motif. Further, in all other eukaryotes the SQ motif characteristic of H2AX in which the serine is phosphorylated requires an adjacent acidic residue (glutamic or aspartic acid) that follows the SQ, a carboxy terminal hydrophobic residue, and an invariant position with regard to the carboxyl terminus [37]. Since the bdelloid SQ sequences lack these defining characteristics, we may conclude that there is no H2AX variant in either bdelloid species. It therefore appears that H2AX is dispensable for bdelloid DNA DSB repair, representing an extraordinary exception to the ubiquity of H2AX across eukaryotes.

Although the functional significance of the unusual features of bdelloid H2A histone variants has not yet been investigated experimentally, the most plausible explanation of our findings is that they have evolved from canonical H2A as part of the ensemble of adaptations that have allowed bdelloid rotifers to survive desiccation and its attendant burden of DNA damage. One may speculate that differences in the conditions and possible nature of DNA breakage may have driven the evolution of different ensembles of H2A variants among eukaryotes. Such an explanation emphasizes the apparent evolutionary flexibility of H2A and its variants as compared to other histone genes and leads to the evolutionary question as to how H2AX and H2A variants, like those found in bdelloids, are reinvented in the mold of canonical H2A [29].

## Materials and Methods

### Genomic Studies

Fosmid genomic libraries were constructed from sheared genomic DNA of the bdelloid species *Adineta vaga* by J. Hur and *Philodina roseola* by K. Van Doninck and P. Wang. Genomic DNA was extracted from purified bdelloid eggs as described previously [4],[53] except that CsCl density-gradient purification was replaced by phenol∶chloroform extraction. The Epicentre CopyControl Fosmid Library Production Kit (EPICENTRE Biotechnologies) was used to construct fosmids of each bdelloid species as previously described [30].

PCR-derived histone probes, using primers based on highly conserved regions of H3 and H2A, were used to screen the genomic libraries of bdelloid rotifers. Each library of each of the above mentioned bdelloid rotifer species was separately hybridized with probes for H3 and H2A.The DNA of all the selected histone fosmids was extracted manually [54] and tested by PCR and direct sequencing of fosmid templates to confirm the presence or absence of each histone gene H3, H4, H2B and H2A, and to verify which type of H2A gene was present. Histone genes were characterized by BLASTX searches on the National Centre for Biotechnology Information non-redundant databases. Exons and introns were mapped by comparison to homologous amino acid sequences using the software *genewise* [55]. Gene prediction and the mapping of introns were also verified using the program *genemark* self-trained on the *C. elegans* genome [56] and the translation tool *Expasy*.

The canonical H2A primers used to make the probes for the bdelloid rotifers could also be used to amplify all H2A genes of the monogonont rotifer *B. plicatilis* containing a canonical H2A core.

For both bdelloid species, four fosmids containing the histone clusters and each copy of canonical H3 were selected for complete sequencing (∼35 kb). DNA from each of these fosmids was purified using Nucleobond Purification kits (BD Biosciences), sheared to a size range of 3–5 kb with a Genemachines Hydroshear (Genomic Solutions) and subcloned in TOPO vectors (Invitrogen) for shotgun sequencing. The resulting sequences were assembled into single contigs as described in [31] and the complete detailed annotation is published in a separate paper [30].

The multiple alignments of the H2A genes and their variants were done using the online version MAFFT v6 [50],[51] with the BLOSUM62 matrix. The “Mafft-homologs” option was enabled only for the alignments including less than 30 sequences. The algorithms used were *G-INS-i* and *L-INS-i* when macroH2A sequences were respectively excluded or included in the alignment. To determine which model of protein evolution would best fit our data we used ProtTest v1.4 [57]. The phylogenetic analyses were carried out with the maximum likelihood approach as implemented in the online version of Bootstrap Raxml [52] available on the CIPRES Portal (http://www.phylo.org/sub_sections/portal/). The following parameters were applied: Dayhoff substitution matrix (selected by ProtTest), empirical base frequencies, maximum likelihood search and 1,000 bootstrapping runs. The trees obtained were displayed using FigTree v1.1.2 (http://tree.bio.ed.ac.uk/software/figtree).

The bdelloid *H2Abd1* amino-acid carboxy-terminus tails or the nucleotide sequences beyond the stop codon were also aligned using MAFFT and displayed as Logos with the interface Weblogo [58],[59] to emphasize the conserved motifs. The Logos format is a graphical representation of aligned sequences where the size of the letter is proportional to the frequency of that particular residue in that position.

For the sliding window analysis, coding sequences of histone genes were first aligned according to their translated peptide sequences with *RevTrans 1.4* [60] using the Dialign 2 method. Sliding windows of the Ka/Ks ratio, among histone genes from the same co-linear pair but in different species, were then generated with *DnaSP 4.0* [61] using 50 bp windows and 10 bp steps.

To measure the nature and magnitude of natural selection acting on bdelloid H2A genes, an amino acid based nucleotide alignment using MAFFT and a corresponding phylogenetic tree with Bootstrap Raxml were built and then used in the program SELECTON for a so-called “High-precision” analysis (http://selecton.tau.ac.il/) [42],[43]. This program evaluates the dN/dS ratio (ω) [46]. Neutral evolution predicts an ω = 1 whereas significantly higher and lower values than 1 are respectively interpreted as evidence for positive and purifying selection. Furthermore, we used PAML v4.1 [44],[45] to test for positive selection along sequences and branches. PAML tests different codon substitution models and performs a likelihood ratio test of positive selection based on the dN/dS ratio. We tested branch-specific selection for every internal branch in the tree. Site-specific selection was tested by comparing different models: “M0” which corresponds to a single dN/dS ratio along the sequence, “M1a” and “M7” for neutral evolution (dN/dS = 1) and “M2a” and “M8” for positive selection (dN/dS>1).

### Proteomic Studies

Histone proteins were extracted from *A. vaga*, *P. roseola* and *B. plicatilis* embryos following a modified protocol of Tops et al. [62]. The *A. vaga* and *P. roseola* rotifer cultures were bleached to obtain clean bdelloid eggs and embryos. *B. plicatilis* embryos were obtained from a filtered, snap-frozen *B. plicatilis* biomass (received from Terry Snell) washed with 0.1% SDS and then bleached. After the bleach treatment, the clean bdelloid and monogonont embryos were washed with extract buffer (10 mM HEPES, pH 7.1; 5 mM MgCl_2_, 2 mM DTT, 10% glycerol and complete protease inhibitor tablets (Roche)) and finally resuspended in 0.5 volume extract buffer. The suspension was dripped in N_2_ (l) and the resulting frozen egg balls were ground in a cold mortar. The obtained powder was thawed on ice and sheared using a chilled dounce homogenizer (30 strokes, tight pistol). The obtained crude extract was centrifuged for 10 min at 14000 rpm to separate the pellet (with nuclei and membranes) from the soluble cytosol. The pellet was washed twice with extract buffer and subsequently resuspended in extract buffer with 0.4N sulfuric acid and left at 4°C overnight. After centrifugation at 14000 rpm for 10 min at 4°C, the acid soluble histone proteins in the supernatant were precipitated with 20% Trichloroacetic acid (TCA). The obtained histone protein pellet was dried 10 min at 95°C and kept at −20°C or immediately resuspended in 1× alkaline sample buffer, heated at 100°C for 10 min and separated by electrophoresis on 15% Tris-glycine SDS-polyacrylamide gels as previously described [63]. Human cells were obtained from the Maniatis laboratory (MCB, Harvard University), washed with extract buffer (as above) and finally resuspended in 0.5 volume extract buffer. The following steps of the histone protein extraction of human cells were identical to the one described for bdelloid and monogonont embryos.

The Coomassie blue stained bands of bdelloids and monogononts on the SDS-PAGE gels were excised (from the lowest band of the gel up to ∼25 kD) and washed with 50% acetonitrile in water. Histones in gel slices were digested with trypsin and subjected to microcapillary reverse-phase HPLC nano-electrospray tandem mass spectrometry (LC-MS/MS) on an LTQ-Orbitrap mass spectrometer (ThermoFisher, San Jose CA). Acquired MS/MS spectra were then correlated with public sequences using the algorithm SEQUEST and custom programs developed in house. The MS/MS peptide sequences were then reviewed in detail for consensus with known proteins and the results manually confirmed for fidelity. The histone protein extractions, SDS-PAGE gels and mass spectrometry analyses of specific bands were done twice for each rotifer. In addition, *de novo* sequencing and additional LC-MS/MS analyses against the translated sequences known for bdelloid and monogonont histones were performed with alternative proteolytic enzymes (chymotrypsin, pronase and elastase) to extend the overall coverage of the carboxyl terminal tails of the histone H2A variants. Exhaustive coverage and redundant acquisition of peptides was maximized by the in-house program Enzyme Optimizer designed to choose a multi-enzyme strategy based on proteotypic peptide properties.

### Accession Numbers

DNA sequences have been deposited at Genbank under accession numbers EU652315 to EU652318, EU850438 to EU850441 and EU853685 to EU853700.

## Supporting Information

Figure S1 Histone gene clusters of bdelloid rotifers *Philodina roseola* (Pr) and *Adineta vaga* (Av). The histone gene cluster is organized as two co-linear pairs A (red) and B (blue). The amino acid sequence of canonical H3, H4 and H2B of both species are aligned and represented as a Logos format (note the conserved sequence among pairs and species).
(1.23 MB TIF)

Figure S2 Mass-spectrometric analysis of the H2A variant protein of *Philodina roseola*. An additional LC-MS/MS analysis was performed to obtain an overall coverage of the carboxyl terminal tail of histone H2A extracted from band H2Av of *Philodina roseola* (Figure 2). The acquired MS/MS spectra are correlated with the sequences of the different H2A genes found in Pr: peptides of the four copies of the histone H2A variant *H2Abd1* were found.
(0.15 MB PDF)

Table S1 Histone gene characteristics of the bdelloid rotifers *Philodina roseola* and *Adineta vaga*. The following histone gene features are given: presence/absence and the number of introns (in brackets); presence of a conserved stem-loop motif and putative HDE element beyond the STOP codon; the mass of each bdelloid histone gene calculated with PeptideMass (in kDalton), a range of masses for the histone genes of animals; the number of amino acids in the C-terminal tail for the bdelloid H2A variants, a maximum number is given for the canonical H2A and H2AX variants in other eukaryotes. The unique bdelloid histone gene features are in bold.
(0.02 MB XLS)
