# Supplementary figures and images for: Phylogenomics of Unusual Histone H2A Variants in Bdelloid Rotifers
Source: PLoS Genet. 2009 Mar 6;5(3):e1000401. doi: 10.1371/journal.pgen.1000401 (PMC2642717; doi:10.1371/journal.pgen.1000401)

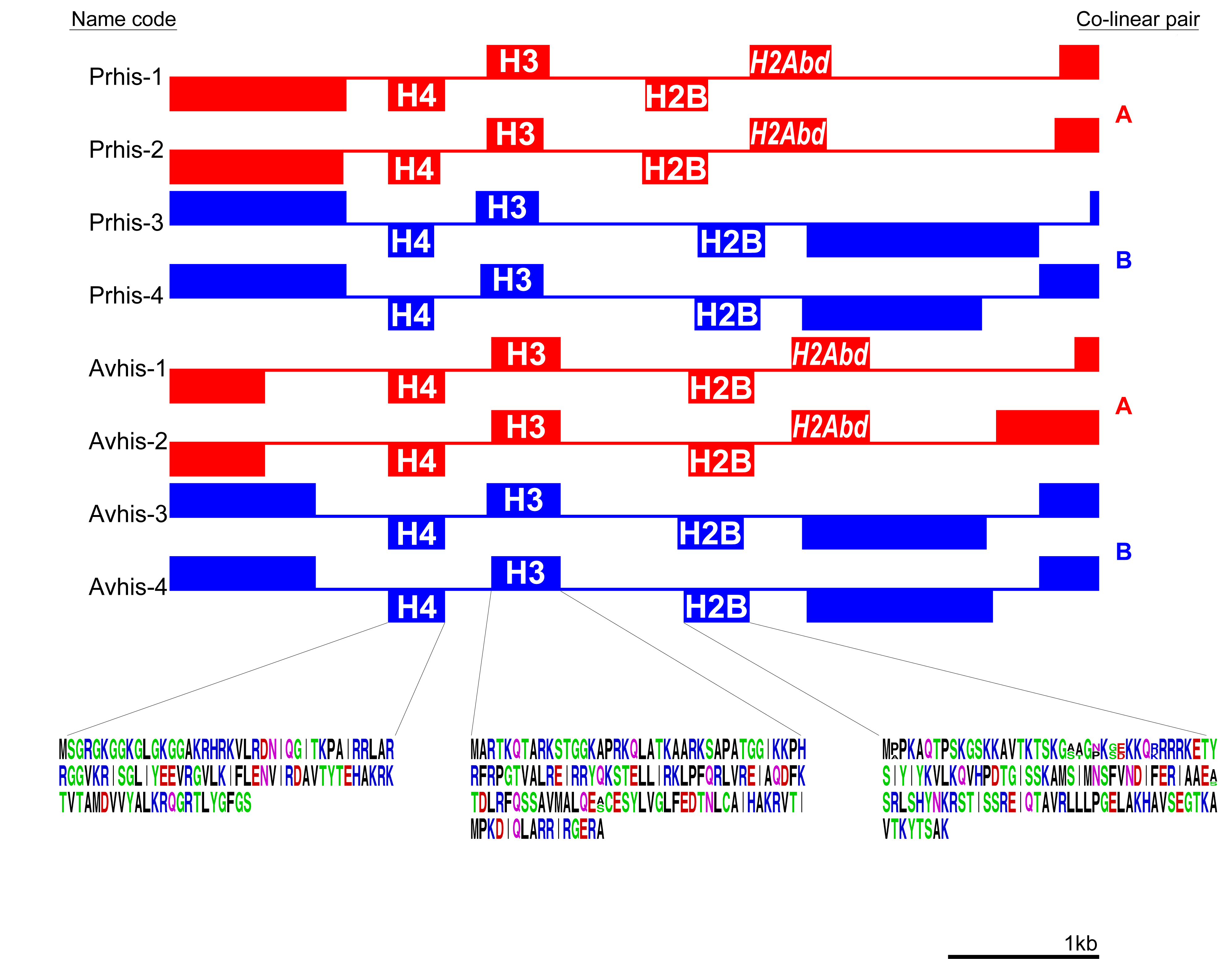

Supplement: Figure S1 — Histone gene clusters of bdelloid rotifers Philodina roseola (Pr) and Adineta vaga (Av). The histone gene cluster is organized as two co-linear pairs A (red) and B (blue). The amino acid sequence of canonical H3, H4 and H2B of both species are aligned and represented as a Logos format (note the conserved sequence among pairs and species). (1.23 MB TIF) [file pgen.1000401.s001.tif]
