# Supplementary material for: Phylogenomics of Unusual Histone H2A Variants in Bdelloid Rotifers
Source: PLoS Genet. 2009 Mar 6;5(3):e1000401. doi: 10.1371/journal.pgen.1000401 (PMC2642717; doi:10.1371/journal.pgen.1000401)
Supplement: Figure S2 — Mass-spectrometric analysis of the H2A variant protein of Philodina roseola. An additional LC-MS/MS analysis was performed to obtain an overall coverage of the carboxyl terminal tail of histone H2A extracted from band H2Av of Philodina roseola (Figure 2). The acquired MS/MS spectra are correlated with the sequences of the different H2A genes found in Pr: peptides of the four copies of the histone H2A variant H2Abd1 were found. (0.15 MB PDF) [file pgen.1000401.s002.pdf]

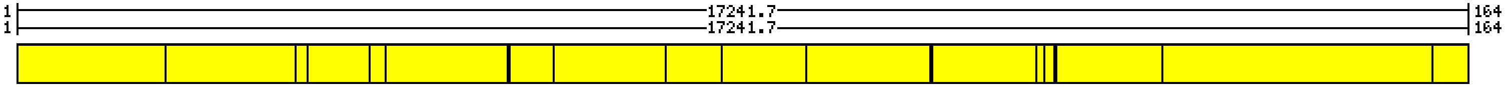

1 MSGRGKTAGS GKARAKAKTR SSRAGLQFPV GRIHRLLRRG NYAERVGAGA PVYLGAVLEY LSAEILELAG NAARDNKKTR

81 IIPRHLQLAI RNDEELNKLL SGVTIAQGGV LPNIQAILLP KKTGPDGAPI STPRETTSAP PKKKSADKGE KSTPSSGAKA

161 SEKA

1 —— 17328.8 —— 165
1 —— 17328.8 —— 165

1 MSGRGKTAGS GKARAKAKTR SSRAGLQFPV GRIHRLLRRG NYAERVGAGA PVYLGAVLEY LSAEILELAG NAARDNKKTR

81 IIPRHLQLAI RNDEELNKLL SGVTIAQGGV LPNIQAILLP KKTGPDGAPI STPRETTSSA PPKKKSADKG EKSTPSSGAK

161 ASEKA

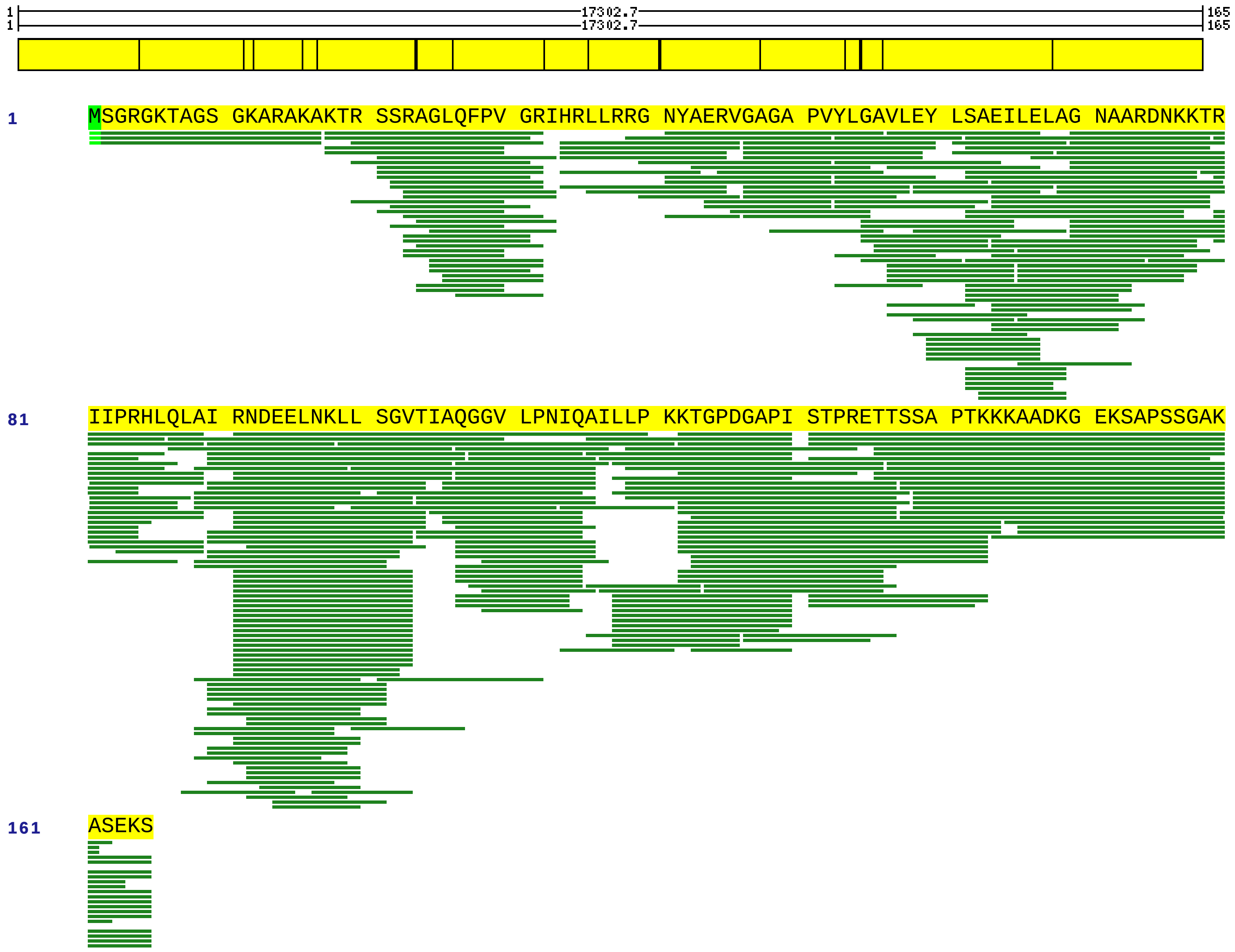
1 17302.7 165
1 17302.7 165
1 MSGRGKTAGS GKARAKAKTR SSRAGLQFPV GRIHRLLRRG NYAERVGAGA PVYLGAVLEY LSAEILELAG NAARDNKKTR
81 IIPRHLQLAI RNDEELNKLL SGVTIAQGGV LPNIQAILLP KKTGPDGAPI STPRETTSSA PTKKKAADKG EKSAPSSGAK
161 ASEKS

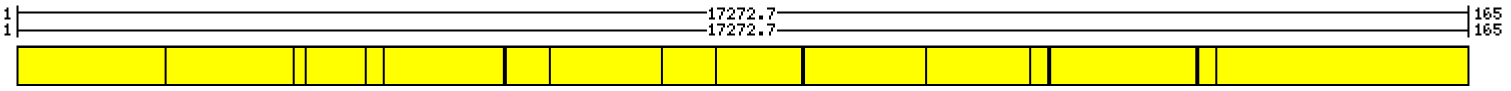

1 MSGRGKTAGS GKARAKAKTR SSRAGLQFPV GRIHRLLRRG NYAERVGAGA PVYLGAVLEY LSAEILELAG NAARDNKKTR

81 IIPRHLQLAI RNDEELNKLL SGVTIAQGGV LPNIQAILLP KKTGPDGAPI STPRETTSSA PPKKKAADKG EKSAASSGAK

161 ASEKS
